# Clinical and epidemiological correlates of treatment change in patients with NMOSD: insights from the CIRCLES cohort

**DOI:** 10.1007/s00415-022-11529-6

**Published:** 2022-12-24

**Authors:** Shervin Gholizadeh, Alex Exuzides, Katelyn E. Lewis, Chella Palmer, Michael Waltz, John W. Rose, Anna Marie Jolley, Jacinta M. Behne, Megan K. Behne, Terrence F. Blaschke, Terry J. Smith, Jennifer Sinnott, Lawrence J. Cook, Michael R. Yeaman, The Guthy-Jackson Charitable Foundation CIRCLES Study Group, Ines Aguerre, Lilyana Amezcua, Tanuja Chitnis, Jessica Coleman Lewis, Casey Engel, May H. Han, Eric C. Klawiter, Alexandra Kocsik, Mason Kruse-Hoyer, Libby Levine, Michael Levy, Melanie Marcille, Maureen A. Mealy, Stephanie Moore, Devin S. Mullin, Katherine E. Nelson, Kaho B. Onomichi, Sarah M. Planchon, Ana Pruitt, Pavle Repovic, Claire S. Riley, Zoe Rimler, Andrew W. Russo, Collin Tanchanco Ocampo, Anna J. Tomczak

**Affiliations:** 1 Genentech, Inc, South San Francisco, CA USA; 2 University of Utah School of Medicine, Salt Lake City, UT USA; 3 The Guthy-Jackson Charitable Foundation, Beverly Hills, CA USA; 4 Departments of Medicine and of Molecular Pharmacology, Stanford University School of Medicine, Stanford, CA USA; 5 University of Michigan Kellogg Eye Center, Ann Arbor, MI USA; 6 Department of Statistics, The Ohio State University, Columbus, OH USA; 7 Geffen School of Medicine at UCLA, Los Angeles, CA USA; 8 Division of Molecular Medicine, David Geffen School of Medicine at UCLA, Institute for Infection and Immunity, Harbor-UCLA Medical Center, Lundquist Institute at Harbor-UCLA Medical Center, 1124 West Carson Street, Torrance, CA 90502 USA

**Keywords:** Neuromyelitis optica spectrum disorder, Treatment, AQP4, Relapse, Demographics

## Abstract

**Objective:**

Neuromyelitis optica spectrum disorders (NMOSD) represent rare autoimmune diseases of the central nervous system largely targeting optic nerve(s) and spinal cord. The present analysis used real-world data to identify clinical and epidemiological correlates of treatment change in patients with NMOSD.

**Methods:**

CIRCLES is a longitudinal, observational study of NMOSD conducted at 15 centers across North America. Patients with ≥ 60 days of follow-up and receiving on-study maintenance treatment were evaluated. The mean annual relapse rate (ARR) was estimated using negative binomial models; the likelihood of treatment change was estimated using Cox proportional hazards models. Relapses were included as time-varying covariates to estimate the relationship to treatment change.

**Results:**

Of 542 patients included, 171 (31.5%) experienced ≥ 1 relapse on the study and 133 patients (24.5%) had ≥ 1 change in the treatment regimen. Two categories of variables significantly correlated with the likelihood of treatment change: (1) relapse: any on-study relapse (hazard ratio [HR] = 2.91; *p* < 0.001), relapse phenotypes (HR range = 2.15–5.49; *p* < 0.001), and pre-study ARR > 0.75 (HR 2.28; *p* < 0.001); 2) disease phenotype: brain syndrome only vs transverse myelitis involvement at onset (HR 2.44; *p* = 0.008), disease duration < 1 vs > 5 years (HR 1.66; *p* = 0.028), or autoimmune comorbidity (HR 1.55; *p* = 0.015). A subset of these factors significantly correlated with shorter time to first rituximab discontinuation.

**Conclusions:**

In CIRCLES, relapse patterns and disease phenotype significantly correlated with changes in the maintenance treatment regimen. Such findings may facilitate the identification of patients with NMOSD who are likely to benefit from treatment change to reduce relapse risk or disease burden and enhance the quality of life.

**Supplementary Information:**

The online version contains supplementary material available at 10.1007/s00415-022-11529-6.

## Introduction

Neuromyelitis optica spectrum disorders (NMOSD) encompass rare autoimmune disease of the central nervous system characterized by inflammation and demyelination, which most frequently affect the optic nerve(s) and spinal cord [1–3]. NMOSD are commonly categorized based on the presence or absence of circulating aquaporin 4 or myelin oligodendrocyte glycoprotein autoantibodies (anti-AQP4-IgG or anti-MOG-IgG, respectively). Approximately 80% of patients diagnosed with NMOSD have detectable anti–AQP4-IgG [4]. NMOSD can substantially impact physical health, with symptoms such as loss of mobility, visual impairment, bowel/bladder dysfunction, and chronic pain, which exert a major negative impact on quality of life [5].

Recently, three biologic therapies (eculizumab [anti-complement protein 5 [C5]; inebilizumab [anti-CD19], and satralizumab [anti-IL-6 receptor]) have shown efficacy in prospective, randomized, placebo-controlled clinical trials at reducing and/or preventing relapse in adults with anti–AQP4-IgG + NMOSD [6]. Even so, maintenance therapies that have not received regulatory approval are still widely used to treat NMOSD, including azathioprine, mycophenolate mofetil, oral steroids, tocilizumab, and rituximab [7, 8].

Open-label studies suggest that several therapies reduce the risk of relapse in patients with NMOSD. For example, the efficacy of rituximab in reducing the annual relapse rate (ARR) has been reported in multiple studies [9–23]. Meta-analyses of such studies found that, collectively, ≈ 60% of patients were relapse-free on rituximab [24]. Serious adverse events such as serious or severe infection or infusion reaction occurred in 26% of patients, raising concerns regarding rituximab as first-line therapy [23, 25]. Importantly, the majority of these studies did not use the 2015 International Panel for NMOSD Diagnosis (IPND) criteria for inclusion. A more recent open-label study of commonly-used therapies found that azathioprine, mycophenolate mofetil, and rituximab, respectively, achieved 54.5%, 60%, and 65% relapse-free effectiveness in a prospective cohort of 72 patients with NMOSD [7]. By comparison, the recently approved therapies for NMOSD implemented standardized relapse definitions with objective adjudication and demonstrated relapse-free rates averaging ≈ 80–94% [26–28]. A recent small randomized controlled study of rituximab (RIN-1) in patients defined as having mild NMOSD demonstrated a 36.8% relative reduction in relapse risk as compared with a steroid comparator [29]. In parallel, a recent open-label study of tocilizumab in a small number of patients with NMOSD (TANGO) [30] applied the IPND criteria and reported a 26% reduction in relapse risk compared with azathioprine. As with rituximab studies, TANGO suggested relatively high rates of treatment-associated adverse events. The above studies used various definitions of *relapse*, were unmasked to investigators and patients (other than RIN-1), did not include trial extensions for long-term evaluation, and were not designed for regulatory review. Thus, despite meritorious efforts, the impact of such agents on disease activity and quality of life has not been studied in regulatory-approved, fully masked, prospective and randomized clinical trials.

Factors that specifically impact treatment decisions in NMOSD are not well understood. Changes in treatment may be prompted by relapses, which signify uncontrolled disease activity and may lead to permanent cumulative disability [31]. In addition, demographic and other epidemiological features may further influence the disease course and the observed relapse profile, which in turn may affect decisions to change the maintenance treatment regimen. The Collaborative International Research in Clinical and Longitudinal Experience Study (CIRCLES) program collected longitudinal clinical data and biospecimens from patients with NMOSD, with goals of understanding disease mechanisms and risks of relapse and facilitating improved patient outcomes and quality of life through advances in diagnosis, relapse prevention, and cures [6].

Initiating effective immunosuppressive therapy as early as possible is essential for preventing relapses and preserving the quality of life in patients with NMOSD. The specific objectives of this study sought to characterize clinical and epidemiological factors correlating with a likelihood of treatment change in patients with NMOSD from the CIRCLES cohort. In addition, clinical correlates of rituximab discontinuation were sought given that it is the most commonly used therapeutic in the CIRCLES cohort. Because the definition of *relapse duration* in NMOSD is often based on clinician assessment—which is subject to variability—this study also applied sensitivity analyses to evaluate how clinician judgment or defined durations of relapse affected correlates of treatment change.

## Methods

### Patients

CIRCLES is a longitudinal, observational study during which participant enrollment and follow-up spanned from March 2013 to March 2020. It comprises three participant cohorts: patients with diagnosed NMOSD, patients with comparative autoimmune or non-autoimmune diseases other than NMOSD, and healthy controls [6]. In the present study, patients included in the analysis were part of the CIRCLES NMOSD cohort, having a clinical diagnosis according to either Wingerchuk 2006 [32] or IPND 2015 [33] criteria, and were classified with respect to anti–AQP4-IgG serostatus [6]. Enrollment was conducted in accordance with guidelines specified by the Office of Human Research Protections of the US Food and Drug Administration (FDA). A standardized protocol, manual of operations, patient study file, and informed consent or assent documents were approved by institutional review boards or respective clinical study sites. Written and verbal consent or assent were obtained before beginning study procedures. The protocol and patient study file were updated periodically.

### Study design

CIRCLES was a prospective, multicenter, cross-sectional, observational and longitudinal study conducted at 15 medical centers across North America enabling the standardized collection of clinical data and biospecimens from patients with NMOSD. Clinical data and biospecimens were obtained from 2013 to 2020 at 6-month intervals and, when possible, proximate to (e.g. within 30 days of) neurologist-confirmed relapses when available. Patients with ≥ 60 days of follow-up and on-study maintenance treatment were evaluated. Patients who had received ≥ 2 doses of rituximab during their participation in the CIRCLES study were included in the analysis of time to first rituximab discontinuation. The total length of follow-up for the entire CIRCLES cohort was up to 7 years (March 2013 to March 2020). The average length of follow-up for patients diagnosed with NMOSD was 2.22 years (range 0.22–6.26).

### Statistical analysis

On-study relapses were noted at their onset date. At the present time, outside the bounds of formal clinical trials, there is no standardized definition of relapse used in routine clinical practice. Relapse events are typically based on subjective clinical judgement, the emergence of new or worsening symptoms consistent with NMOSD, and/or evidence of lesions on imaging. Consistent with established clinical practices, the defined duration for all relapses was set to 90 days [34–39]. Therefore, to harmonize relapse history and minimize the impact of recall bias or variability, relapses that began within 90 days before study consent were counted as on-study relapses, and those starting > 90 days prior to consent were considered pre-study relapses. Sensitivity analyses were performed in which relapses were counted as on-study if they began within 30 days before the study start (and those starting longer than 30 days prior to consent considered pre-study) and in which relapses were counted as on-study if they began within the clinician judgment window before study consent. The mean ARR was estimated using negative binomial models. Treatment change was defined as any change in the treatment regimen, which could include changing from one drug to another or adding or removing a drug from an existing regimen. HRs for time to first treatment change were estimated using univariate Cox proportional hazards models for patient demographic and clinical features. These features included sex, race/ethnicity, disease duration, age at disease onset, anti–AQP4-IgG serostatus, disease onset and relapse phenotypes (optic neuritis; transverse myelitis; area postrema syndrome; brainstem syndrome; and/or brain/cerebral syndrome), pre-study (self-reported) ARR, and autoimmune comorbidities. The relationship between relapse and treatment change was evaluated by including relapses as time-dependent variables; one analysis evaluated the association with experiencing any on-study relapse, while a second analysis evaluated how different relapse phenotypes related to the likelihood of treatment change (relative to no relapse). Among patients who had received ≥ 2 doses of rituximab, the relationship between clinical and epidemiological factors and time to first rituximab discontinuation (TFRD) was evaluated (same variates as listed above plus specific autoimmune categories). Rituximab discontinuation was defined as (1) no re-dosing of rituximab within 1 year and (2) initiation of a new maintenance therapy.

## Results

### Patients

Patients from the CIRCLES NMOSD cohort (*n* = 542) were included in this analysis. Patients were predominantly female (86.5%), White (57.4%), and seropositive for anti–AQP4-IgG (83.9%) (Table 1). The majority of patients (72.2%) were aged ≥ 30 years at disease onset, with a disease duration of ≥ 1 year (81.9%). One hundred fifty-three (28.2%) patients had an autoimmune comorbidity in addition to NMOSD: including lupus (6.8%), Sjögren’s syndrome (6.3%), rheumatoid arthritis (2.8%), and myasthenia gravis (2.0%). At the time of study consent, 42.4% of patients were receiving rituximab, 17.0% mycophenolate mofetil, 12.4% low-dose maintenance steroids, 11.8% azathioprine, and 0.7% tocilizumab. Disease onset phenotype included transverse myelitis (TM) or TM + brain involvement (BR) in 35.6% of patients, optic neuritis (ON) or ON + BR in 32.7%, through symptoms alone in 14.6%, ON + TM or ON + TM + BR in 10.7%, and BR only in 6.4%.

**Table 1:** Patient demographics and clinical characteristics

Clinical/Epidemiological Factor	Patients, *n* (%) (Total *N* = 542)
Median length of follow-up (years)	2.22 (0.22–6.26)
Female sex	469 (86.5)
Race/ethnicity	
Asian	47 (8.7)
Black or African American	124 (22.9)
Hispanic or Latino	60 (11.1)
White	311 (57.4)
Anti–AQP4-IgG +	455 (83.9)
Age at disease onset	
< 30 years	151 (27.9)
30–49 years	260 (48)
≥ 50 years	131 (24.2)
Disease duration before study entrance	
< 1 year	98 (18.1)
1–5 years	186 (34.3)
> 5 years	258 (47.6)
Pre-study ARR (self-reported)	
Rate < 0.25	249 (45.9)
Rate 0.25–0.75	166 (30.6)
Rate > 0.75	127 (23.4)
Disease onset phenotype	
ON only and ON + BR	177 (32.7)
TM only and TM + BR	193 (35.6)
ON + TM and ON + TM + BR	58 (10.7)
BR only	35 (6.4)
Confirmed through symptoms	79 (14.6)
Comorbidities	
Any other autoimmune comorbidity	153 (28.2)
Lupus	37 (6.8)
Sjögren syndrome	34 (6.3)
Rheumatoid arthritis	15 (2.8)
Myasthenia gravis	11 (2.0)
Treatment at study start^a^	
Rituximab	230 (42.4)
Mycophenolate mofetil	92 (17.0)
Maintenance steroids	67 (12.4)
Azathioprine	64 (11.8)
Tocilizumab	4 (0.7)

*Anti–AQP4-IgG*, aquaporin 4 autoantibody; *ARR*, annualized relapse rate; *BR*, brain involvement; *ON*, optic neuritis; *TM*, transverse myelitis

^a^Treatment categories are not mutually exclusive. Each is a binary yes/no variable (“yes” level is summarized in this table). It is possible that patients were not on any treatment, multiple treatments, or other treatments on Day 1

### Associations between demographics, clinical phenotype and treatment change

**Fig. 1:**
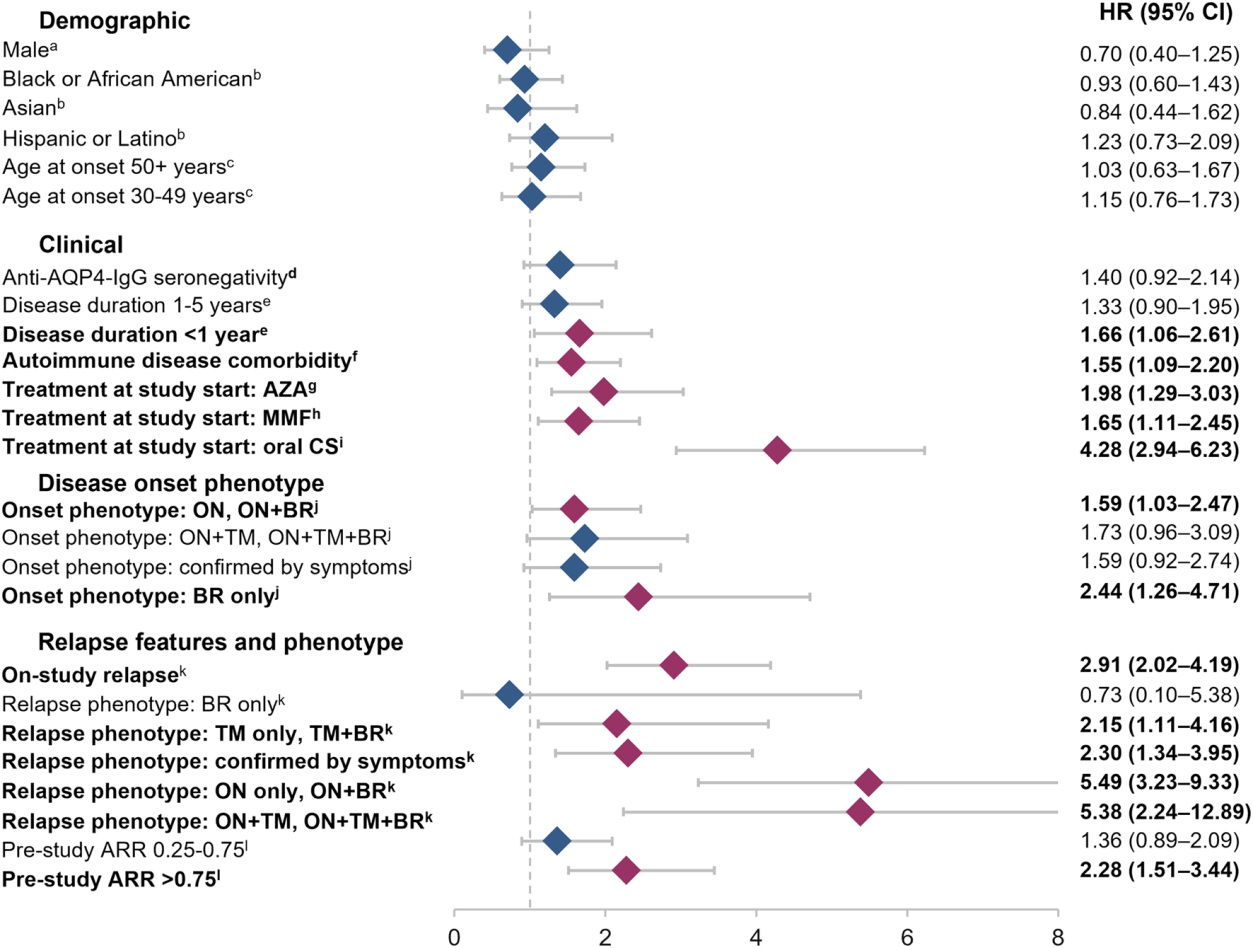
Hazard ratios for time to first treatment change. ^a^Reference female. ^b^Reference White. ^c^Reference < 30 years. ^d^Reference seropositivity. ^e^Reference > 5 years. ^f^Reference no autoimmune disease comorbidity. ^g^Reference no AZA; ^h^Reference no MMF; ^i^Reference no oral CS; ^j^Reference TM, TM + BR. ^k^Reference no prior on-study relapse. ^l^Reference pre-study ARR < 0.25. *anti–AQP4-IgG*, aquaporin-4 autoantibody; *ARR*, annualized relapse rate; *AZA*, azathioprine; *BR*, brain involvement; *CS*, corticosteroids; *HR*, hazard ratio; *MMF*, mycophenolate mofetil; *ON*, optic neuritis; *TM*, transverse myelitis

In the overall study cohort, we examined demographic and clinical phenotype as potential correlates of treatment change. In the core demographic variables studied (age, biological sex, race and ethnicity), no associations were observed relative to the probability of treatment change (Fig. 1). However, clinical phenotypes were found to be associated with treatment change. For example, 133 patients (24.5%) changed treatment at least once on-study. The likelihood of treatment change was significantly associated with disease duration < 1 year (HR [95% CI] 1.66 [1.06–2.61], *p* = 0.028), autoimmune disease comorbidity (1.55 [1.09–2.20], *p* = 0.015), and ON and/or BR at disease onset (ON, ON + BR: 1.59 [1.03–2.47], *p* = 0.037; BR only: 2.44 [1.26–4.71], *p* = 0.008) (Fig. 1).

### Association between relapse features and treatment change

A total of 292 relapses (including those that began within 90 days of the study start) were reported in 171 patients during the study. Of these, 38.4% were confirmed through symptoms, 24.6% involved ON only or ON + BR, 22.8% involved TM only or TM + BR, 8.9% involved ON + TM or ON + TM + BR, and 5.4% involved BR only (Table 2). Having an on-study relapse (HR [95% CI]: 2.91 [2.02–4.19], *p* < 0.001) or pre-study (self-reported) ARR > 0.75 (2.28 [1.51–3.44], *p* < 0.001) were significantly associated with the likelihood of treatment change (Fig. 1). Compared with those not experiencing a relapse, on-study relapse phenotypes that were significantly associated with the likelihood of treatment change included relapses involving ON only or ON + BR (HR [95% CI]: 5.49 [3.23–9.33], *p* < 0.001), relapses involving ON + TM or ON + TM + BR (5.38 [2.24–12.89], *p* < 0.001), relapses involving TM only or TM + BR (2.15 [1.11–4.16], *p* = 0.023), and relapses confirmed through symptoms (2.30 [1.34–3.95], *p* = 0.002). These results were consistent in sensitivity analyses (Supplemental Fig. 1).

**Table 2:** Relapse phenotype frequency

Relapse phenotype	Relapse occurring most proximate to treatment change^a^, *n* (%)
ON only or ON + BR	55 (24.6)
TM only or TM + BR	51 (22.8)
ON + TM or ON + TM + BR	20 (8.9)
BR only	12 (5.4)
Confirmed through symptoms	86 (38.4)

*BR*, brain involvement; *ON*, optic neuritis; *TM*, transverse myelitis

^a^A total of 224 relapse events were reported by patients. Relapses confirmed through symptoms are those judged to be relapses based on clinical symptoms only, in the absence of clinical signs including confirmed TM, ON, or brain/brainstem lesions (i.e. signs requiring imaging confirmation)

### Influence of demographic factors on ARR

**Fig. 2:**
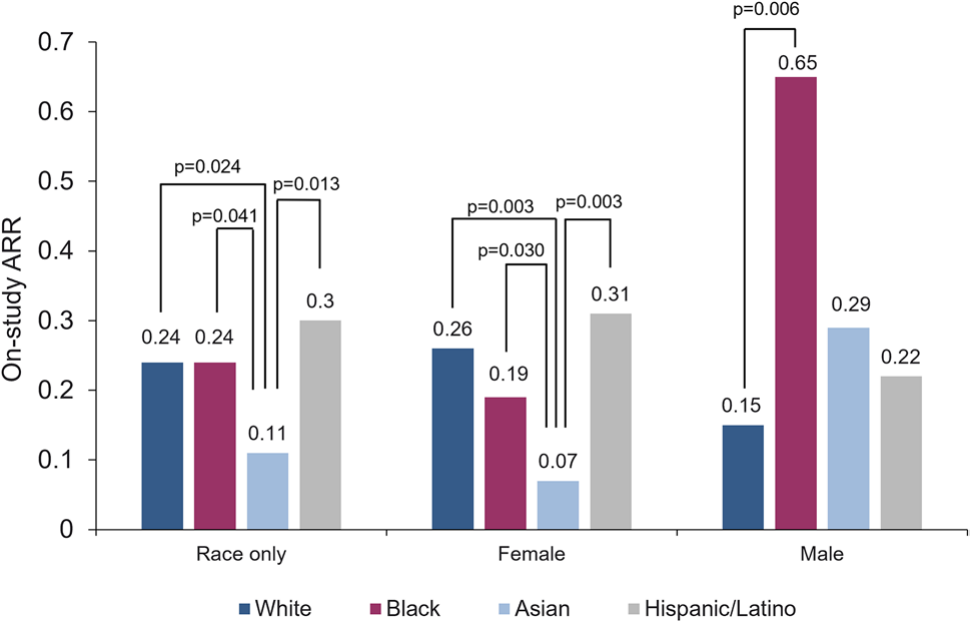
On-study ARR by race/ethnicity and sex. *ARR*, annualized relapse rate

The mean on-study ARR differed by sex and race/ethnicity (Fig. 2). Overall, the mean on-study ARR was significantly higher in Hispanic, White, and Black patients than in Asian patients (0.30 vs 0.11, *p* = 0.013; 0.24 vs 0.11, *p* = 0.024; and 0.24 vs 0.11, *p* = 0.041, respectively). Sex alone did not significantly correlate with on-study ARR; however, stratifying by sex/race/ethnicity revealed some significant differences (Fig. 2 and Table 3). The mean ARR was significantly lower in Asian women than in Hispanic (0.07 vs 0.31, *p* = 0.003), White (0.07 vs 0.26, *p* = 0.003), or Black (0.07 vs 0.19, *p* = 0.030) women. By comparison, the mean ARR was significantly lower in White than in Black men (0.15 vs 0.65, *p* = 0.006). Furthermore, the patterns of ARR when stratified by anti-AQP4 + serostatus vs. the overall study cohort are summarized in Table 3. Here, three significant correlates relative to ARR were identified in the anti-AQP4 + cohort that were not found to be significant in the overall cohort: Latino > White overall (respective ARR 0.34 vs. 0.21; *p* = 0.046); Latino > White female only (respective ARR 0.35 vs. 0.21; *p* = 0.040); and Latino > Black female only (respective ARR 0.35 vs. 0.20; *p* = 0.042).

**Table 3:** *P* values for pairwise comparison of on-study annualized relapse rate

	White (ARR 0.24)	AQP4 + White (ARR 0.21)	Black (ARR 0.24)	AQP4 + Black (ARR 0.22)	Asian (ARR 0.11)	AQP4 + Asian (ARR 0.07)	Latino (ARR 0.30)	AQP4 + Latino (ARR 0.34)
Race only								
White (ARR 0.24)	Ref.		0.912		**0.024**		0.392	
AQP4 + White (ARR 0.21)		Ref.		**0.707**		**0.008**		0.046
Black (ARR 0.24)			Ref.		**0.041**		0.405	
AQP4 + Black (ARR 0.22)				Ref.		**0.007**		0.131
Asian (ARR 0.11)					Ref.		**0.013**	
AQP4 + Asian (ARR 0.07)						Ref.		**0.001**
Latino (ARR 0.30)							Ref.	
AQP4 + Latino (ARR 0.34)								Ref.

Female only	White (ARR 0.26)	AQP4 + White (ARR 0.21)	Black (ARR 0.19)	AQP4 + Black (ARR 0.20)	Asian (ARR 0.07)	AQP4 + Asian (ARR 0.08)	Latino (ARR 0.31)	AQP4 + Latino (ARR 0.35)
White (ARR 0.26)	Ref.		0.197		**0.003**		0.483	
AQP4 + White (ARR 0.21)		Ref.		0.778		**0.020**		**0.040**
Black (ARR 0.19)			Ref.		**0.030**		0.123	
AQP4 + Black (ARR 0.20)				Ref.		**0.036**		**0.042**
Asian (ARR 0.07)					Ref.		**0.003**	
AQP4 + Asian (ARR 0.08)						Ref.		**0.001**
Latino (ARR 0.31)							Ref.	
AQP4 + Latino (ARR 0.35)								Ref.

Male only	White (ARR 0.15)	AQP4 + White (ARR 0.18)	Black (ARR 0.65)	AQP4 + Black (ARR 0.52)	Asian (ARR 0.29)	AQP4 + Asian (ARR 0.00)	Latino (ARR 0.22)	AQP4 + Latino (ARR 0.14)
White (ARR 0.15)	Ref.		**0.006**		0.317		0.651	
AQP4 + White (ARR 0.18)		Ref.		0.062		1.000		0.813
Black (ARR 0.65)			Ref.		0.272		0.241	
AQP4 + Black (ARR 0.52)				Ref.		1.000		0.276
Asian (ARR 0.29)					Ref.		0.785	
AQP4 + Asian (ARR 0.00)						Ref.		1.000
Latino (ARR 0.22)							Ref.	
AQP4 + Latino (ARR 0.14)								Ref.

On-study relapses included those that began up to 90 days before study start

*AQP4* + , anti–aquaporin 4 seropositive; *ARR*, annualized relapse rate

Bold, significant difference at the *P* < 0.05 level

### Association between clinical and epidemiological features and TFRD

**Fig. 3:**
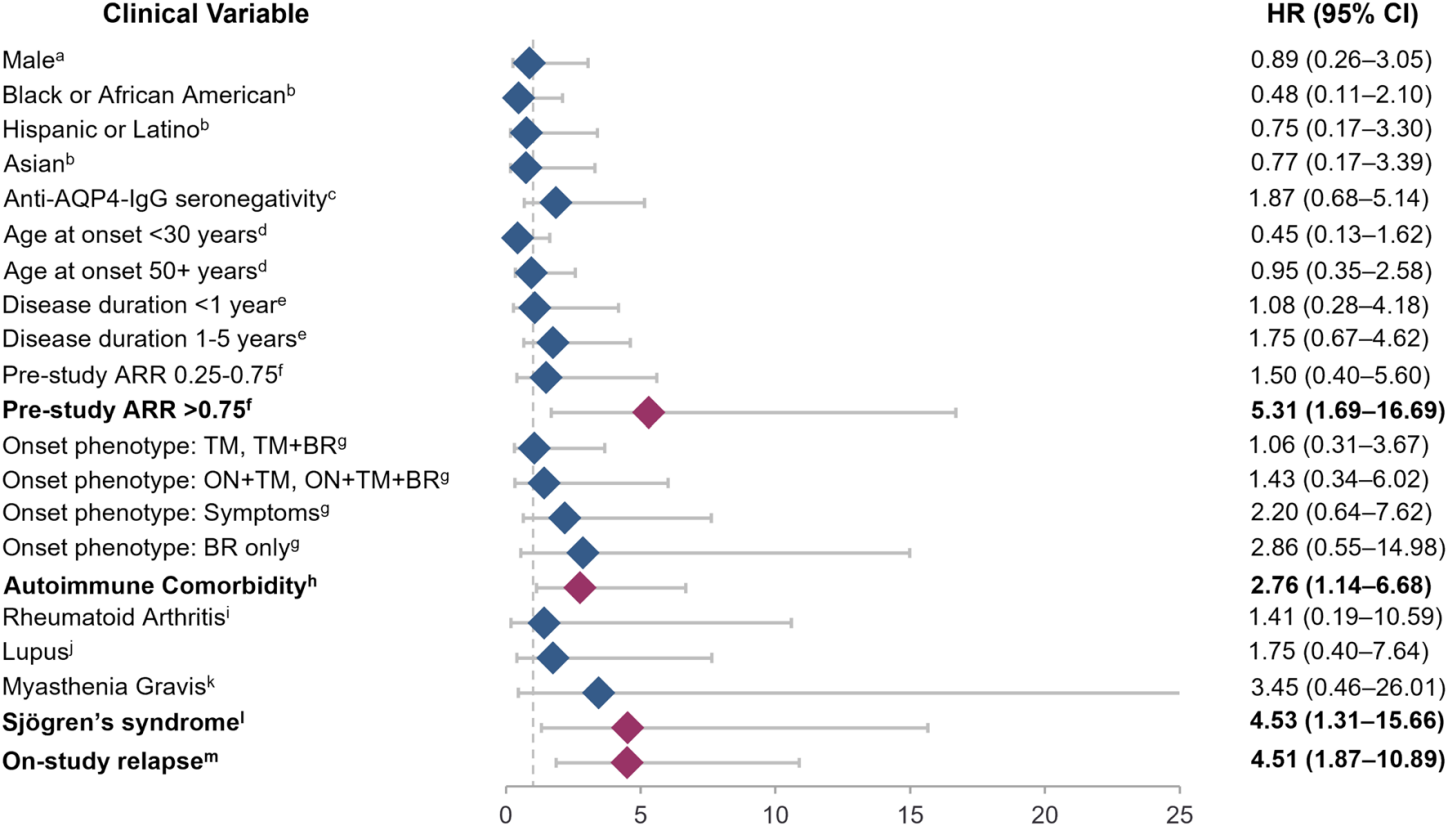
HRs for time to first rituximab discontinuation among patients on rituximab (at least two doses). ^a^Reference female. ^b^Reference White. ^c^Reference seropositivity. ^d^Reference 30–49 years. ^e^Reference > 5 years. ^f^Reference ARR < 0.25. ^g^Reference ON, ON + BR. ^h^Reference no comorbidity. ^i^Reference no rheumatoid arthritis. ^j^Reference no lupus. ^k^Reference no myasthenia gravis. ^l^Reference no Sjögren syndrome. ^m^Reference no on-study relapse. *anti–AQP4-IgG*, aquaporin 4 autoantibody; *ARR*, annualized relapse rate; *BR*, brain involvement; *HR*, hazard ratio; *ON*, optic neuritis; *TM*, transverse myelitis

A total of 320 patients received ≥ 2 doses of rituximab at some point during the study, were being treated with rituximab at the time of enrollment, or began treatment at a later time point. Clinical and epidemiological features of these patients are described in Table 4. Of these patients, 30.6% relapsed at least once on-study, and 14.1% changed treatment regimen at least once on-study. A regimen change included switching to another treatment, switching from rituximab alone to rituximab plus another therapy, or switching from rituximab plus another therapy to rituximab alone. This finding suggests that patients on rituximab are less likely to change therapy than is the overall study population. Overall, 20 individuals (6.3%) discontinued rituximab; accounting for censoring, the probability of discontinuing rituximab within 5 years was estimated to be 24%. Pre-study (self-reported) ARR at the time of enrollment > 0.75 (HR [95% CI] 5.31 [1.69–16.69], *p* = 0.004), having an on-study relapse (4.51 [1.87–10.89], *p* < 0.001), or having any autoimmune comorbidity diagnosis (2.76 [1.14–6.68], *p* = 0.025)—particularly a Sjögren’s syndrome diagnosis (4.53 [1.31–15.66], *p* = 0.017)—were significantly associated with a shorter TFRD (Fig. 3). In the current study, presence of concomitant lupus or myasthenia gravis was not significantly associated with TFRD. These effects were consistent in the sensitivity analyses (Supplementary Fig. 2).

**Table 4:** Patient demographics and clinical and epidemiological factors among patients receiving at least two doses of rituximab

Clinical/epidemiological factor	Patients, *n* (%) (Total *N* = 320)
Female sex	269 (84.1)
Race/ethnicity	
Asian	30 (9.4)
Black or African American	68 (21.3)
Hispanic or Latino	39 (12.2)
White	183 (57.2)
Anti–AQP4-IgG +	269 (84.1)
Age at disease onset	
< 30 years	89 (27.8)
30–49 years	149 (46.6)
≥ 50 years	82 (25.6)
Disease duration before study entrance	
< 1 years	63 (19.7)
1–5 years	116 (36.3)
> 5 years	141 (44.1)
Pre-study ARR (self-reported)	
Rate < 0.25	144 (45.0)
Rate 0.25–0.75	99 (30.9)
Rate > 0.75	77 (24.1)
Disease onset phenotype	
ON only and ON + BR	102 (31.9)
TM only and TM + BR	109 (34.1)
ON + TM and ON + TM + BR	38 (11.9)
BR only	21 (6.6)
Confirmed through symptoms	50 (15.6)
Comorbidities	
Any other autoimmune comorbidity	83 (25.9)
Lupus	18 (5.6)
Sjögren syndrome	16 (5.0)
Rheumatoid arthritis	9 (2.8)
Myasthenia gravis	5 (1.6)
Treatment at study start^a^	
Rituximab	175 (54.7)
Mycophenolate mofetil	19 (5.9)
Maintenance steroids	28 (8.8)
Azathioprine	14 (4.4)
Tocilizumab	0 (0)

*Anti–AQP4-IgG*, aquaporin 4 autoantibody; *ARR*, annualized relapse rate; *BR*, brain involvement; *ON*, optic neuritis; *TM*, transverse myelitis

^a^Treatment categories are not mutually exclusive. Each is a binary yes/no variable (“yes” level is summarized in this table). It is possible that patients were not on any treatment, multiple treatments, or other treatments on day 1

## Discussion

The current study was performed to better understand factors influencing maintenance treatment change in NMOSD. The findings are not intended to be prescriptive regarding any specific therapy or determinant of therapeutic change. Changing maintenance therapy in NMOSD is always an important consideration for patients with inadequate control of disease activity. In addition, patients in whom therapy is associated with serious adverse events (severe or recurring infections, reactions, etc.) may also benefit from a change in maintenance therapy. Thus, goals for therapeutic change include potentially achieving (1) greater efficacy in reducing risk or severity of relapse, (2) greater efficacy in reducing non-relapse disease burden (e.g. pain, incontinence, fatigue, etc.), and (3) greater safety in reducing the risk of adverse events (e.g. infections, reactions, etc.) or in other circumstances (e.g. pregnancy, vascular access concerns, etc.). Three therapies have now received approval from the FDA and equivalent regulatory agencies worldwide based on large randomized, fully masked, prospective and controlled clinical trials. In these trials, each of the approved therapies demonstrated safety and significant efficacy in adult patients diagnosed with NMOSD who have detectable anti-AQP4 autoantibody.

The primary goal of this analysis was to identify clinical and epidemiological factors that correlate with maintenance treatment change in patients with NMOSD receiving therapies such as azathioprine, mycophenolate mofetil, and rituximab. Understanding patterns of treatment change and correlates thereof may offer new insights into potential causal relationships and facilitate predictive capabilities of treatment change. In turn, such knowledge may facilitate the application of therapeutics leading to improved outcomes. Our previous complementary studies [6, 40, 41] have explored bivariate and multivariate relationships among demographic, socioeconomic and related factors that may influence clinical outcomes or quality of life in patients with NMOSD.

Higher probability of treatment change in this cohort of patients with NMOSD was significantly more likely in patients with shorter disease duration, concomitant autoimmune disease, NMOSD disease onset phenotype with ON and/or BR, higher pre-study ARR, and on-study relapse activity. While there were differences in mean ARR associated with sex and race/ethnicity, these factors did not significantly correlate with the probability of treatment change. Shorter TFRD in patients who received rituximab during the study was significantly associated with higher pre-study ARR, on-study relapse activity, and concomitant autoimmune disease (particularly Sjogren’s syndrome). Overall, uncontrolled disease activity, as evident by relapses, emerged as an important factor in predicting treatment change.

In addition to identifying clinical and epidemiological correlates of treatment change, it is also important to understand how the defined duration of relapse influences these correlates. In this analysis, 90 days was set as the defined duration for all relapses; thus, relapses that started within 90 days before study consent were counted as on-study relapses. This definition of duration is consistent with the clinical characterization of relapse onset vs resolution in recent reports [35, 36]. Sensitivity analyses were performed in which the defined duration was set at 30 days or per clinician judgement. These sensitivity analyses demonstrated consistency in the relationship between on-study relapses and treatment change as compared with the 90-day definition. Thus, a relapse window spanning 90 days may represent a reasonable definition for NMOSD research assessing relapse effects.

Until 2019, there were no approved therapies to treat NMOSD; there are now three therapies approved, one of which was repurposed and two that were developed de novo [6]. Although the data in the present study were collected before any approved therapies were available, findings suggest that patients may not seek to proactively change to another therapy pre-emptive of relapse. Likewise, physicians may not recommend switching to another therapy until a patient experiences breakthrough disease. It is possible that patients and/or physicians may prefer treatment that is familiar or for which logistical barriers associated with initiating a new therapy, such as access or reimbursement, have been overcome—even in the absence of FDA approval. Notably, in this study, shorter disease duration was associated with the likelihood of treatment change; patients earlier in their disease course may be more willing to try different therapies. Such an observation may relate to the tendency for disease activity to be greater earlier in the disease course. Thus, understanding relationships between disease course and treatment change may inform or predict the tendency to discontinue one therapeutic and begin another relative to key clinical milestones. In turn, optimizing therapeutic efficacy may translate to reduced disease burden and improved quality of life. The monoclonal antibodies eculizumab, inebilizumab, and satralizumab are biologic therapies that have been approved by the FDA and other regulatory agencies for the treatment of anti–AQP4-IgG + NMOSD in adult patients [6]. Given the efficacy of approved, targeted biologic therapies demonstrated in randomized, prospective controlled trials, patients experiencing suboptimal disease control in terms of relapses, disabling disease burden independent of relapses (e.g. chronic pain, incontinence or fatigue), or those suffering severe or recurring adverse events may benefit from a change in the treatment regimen.

The current focus of immunosuppressive therapy in NMOSD is to reduce the relapse rate and disease severity [42]. Preventing relapses is essential to minimizing the accumulation of permanent disability [31]. While this study examined several clinical characteristics and identified relapse as a major predictor of treatment change, future studies are needed to evaluate how other factors, such as disability and quality of life, affect treatment change. It is also possible that cryptic disease activity in the absence of frank relapses, but which contributes to disability, may also benefit from approved therapeutics. Furthermore, there is a need for an endpoint that accurately measures disability in NMOSD, particularly in the absence of relapses or in between relapses. Understanding how disability affects treatment patterns may provide key insights into aiding the optimization of therapy for patients with NMOSD.

There are multiple considerations to keep in mind when interpreting these data. This study only included a subset of therapies for NMOSD; therefore, future studies will be necessary to understand the impact of recently regulatory-approved biologic therapies on treatment change in NMOSD. Additionally, because there is no standard definition of relapse in NMOSD, clinician judgement may vary and introduce some degree of variance in relapse adjudication. Likewise, newly diagnosed patients with NMOSD will have additional treatment options available and different treatment histories than the cohort described here, which may lead to different results. Importantly, while distinct definitions of relapse windows were analyzed, the degree to which such definitions may impact associations are dependent on the variables under consideration for that analysis. Further, to maintain robust data integrity, treatment history prior to study enrollment was not included in the analyses, as it is often fraught with recall bias and inadvertent inaccuracies. As with any cohort study, the CIRCLES cohort may not be generalizable to a broader population, and the results of this study will need to be verified and reproduced in other patient cohorts. It is unknown from this study whether patients were offered therapies and declined, and the study did not evaluate whether financial concerns were a factor in treatment change.

The results of this study suggest potential clinical correlates of treatment change in patients with NMOSD as the field evolves from non-specific immunosuppressants that have not been evaluated in formal clinical trials to approved biologic therapies. These findings may help facilitate identifying determinants or predictors of treatment change and aid in optimizing therapy to reduce disease burden and enhance quality of life in patients with NMOSD.

## Supplementary Information

Below is the link to the electronic supplementary material.

Supplementary file1 (DOCX 561 kb)

## Data Availability

CIRCLES data used in this study are available by request and agreement with terms of use.

## Abbreviations

anti–AQP4-IgG: Aquaporin 4 autoantibody
ARR: Annual relapse rate
BR: Brain involvement
FDA: US Food and Drug Administration
HR: Hazard ratio
IPND: International Panel for NMOSD Diagnosis
NMOSD: Neuromyelitis optica spectrum disorder
ON: Optic neuritis
TM: Transverse myelitis
