# Supplementary material for: Clinical and epidemiological correlates of treatment change in patients with NMOSD: insights from the CIRCLES cohort
Source: J Neurol. 2022 Dec 24;270(4):2048–58. doi: 10.1007/s00415-022-11529-6 (PMC10025181; doi:10.1007/s00415-022-11529-6)
Supplement: Supplementary file 1 — Supplementary file1 (DOCX 561 kb) [file 415_2022_11529_MOESM1_ESM.docx]

**Supplemental Information**

**Supplementary Fig. 1** Sensitivity analysis HRs for treatment change relative to pre-study relapse inclusion criteria

a. Relapses counted as on-study if they started in the 30 days before study start*


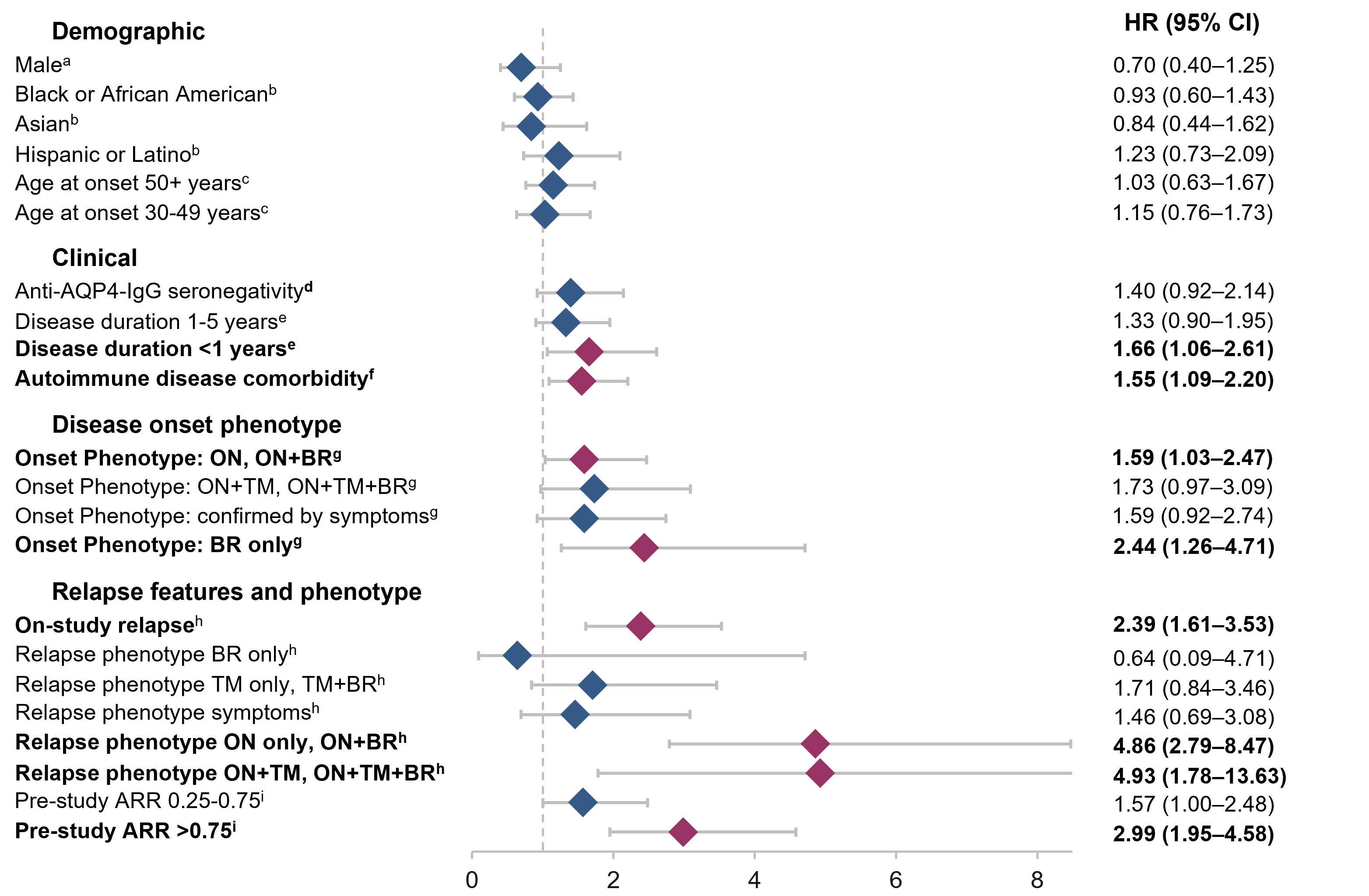


b. Relapses counted as on-study if they started within the clinician judgement window before the study start*

**
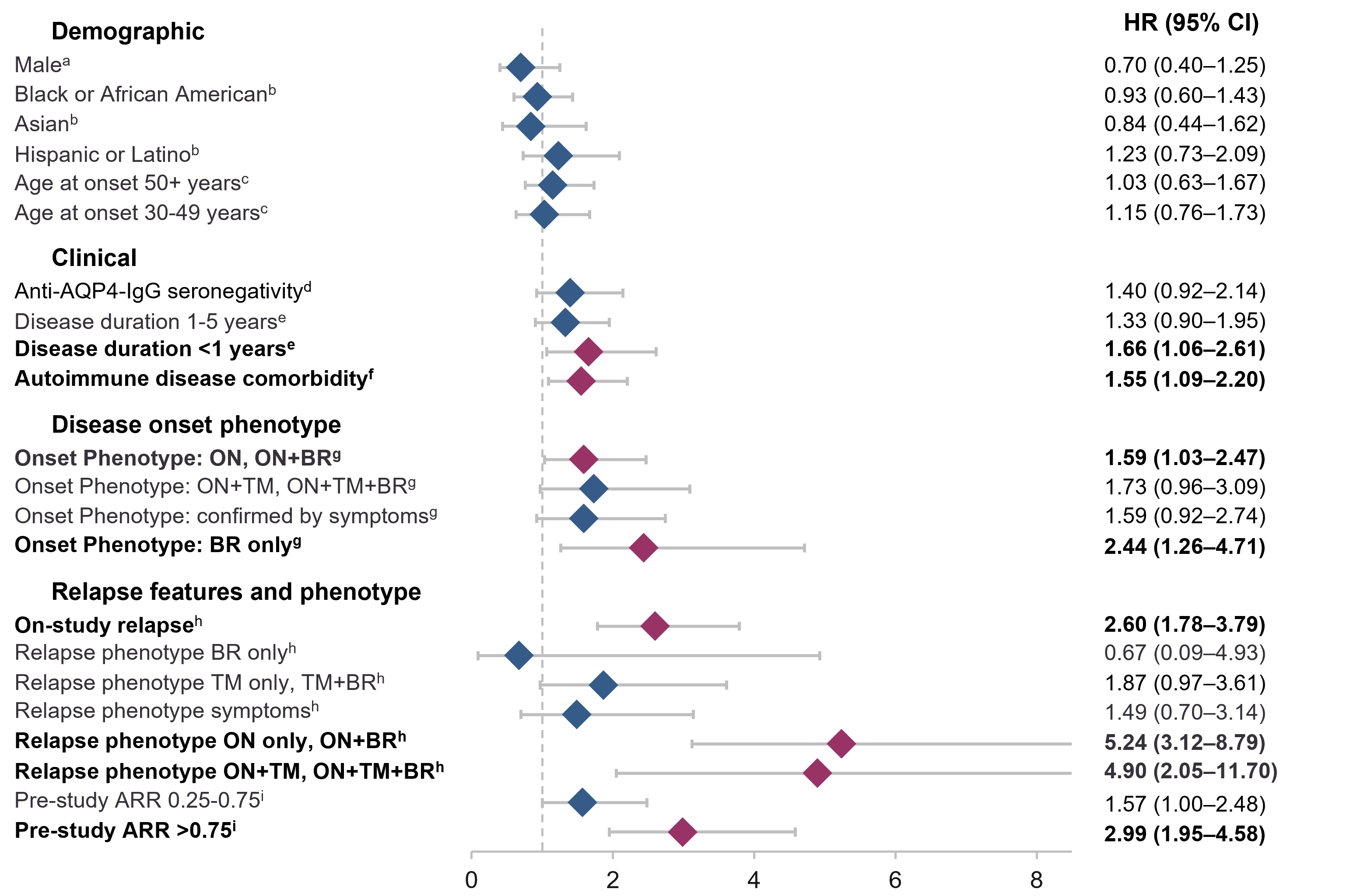
**

*Note: only variables related to on-study relapse/phenotype are subject to change based on the window of relapse definition. ^a^Reference female. ^b^Reference White. ^c^Reference <30 years. ^d^Reference seropositivity. ^e^Reference >5 years. ^f^Reference no autoimmune disease comorbidity. ^g^Reference TM, TM + BR. ^h^Reference no prior on-study relapse. ^i^Reference pre-study ARR <0.25. Anti–AQP4-IgG, aquaporin 4 autoantibody; ARR, annualized relapse rate; BR, brain involvement; HR, hazard ratio; ON, optic neuritis; TM, transverse myelitis

**Supplementary Fig. 2** Sensitivity analysis HRs for rituximab discontinuation among patients taking rituximab (at least two doses) relative to pre-study relapse inclusion criteria

a. Relapses counted as on-study if they started in the 30 days before the study start*


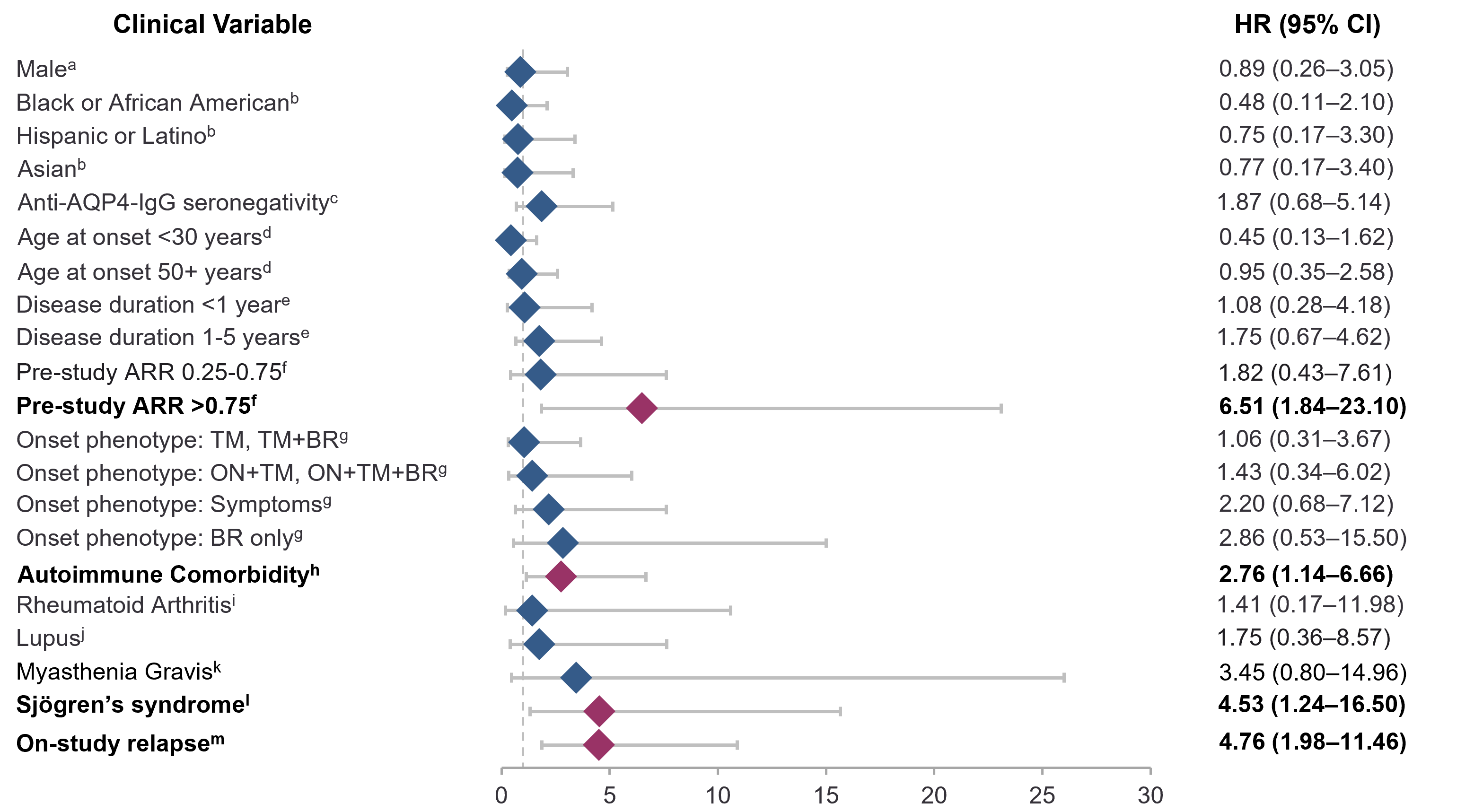


b. Relapses counted as on-study if they started within the clinician judgement window before study start*


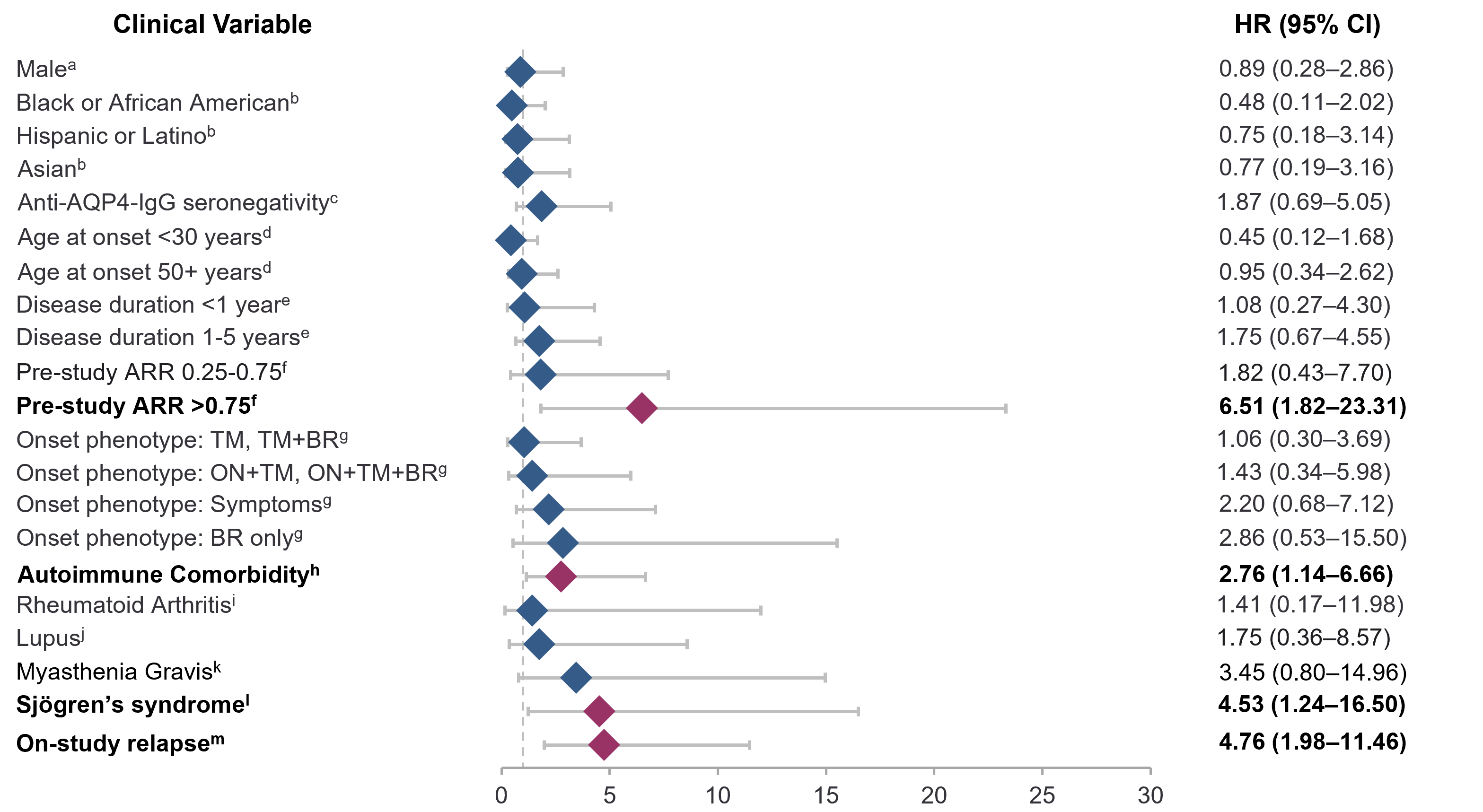


*Note: only variables related to on-study relapse/phenotype are subject to change based on the window of relapse definition. ^a^Reference female. ^b^Reference White. ^c^Reference seropositivity. ^d^Reference 30–49 years. ^e^Reference >5 years. ^f^Reference ARR <0.25. ^g^Reference ON, ON + BR. ^h^Reference no comorbidity. ^i^Reference no rheumatoid arthritis. ^j^Reference no lupus. ^k^Reference no myasthenia gravis. ^l^Reference no Sjögren syndrome. ^m^Reference no on-study relapse. Anti–AQP4-IgG, aquaporin 4 autoantibody; ARR, annualized relapse rate; BR, brain involvement; HR, hazard ratio; ON, optic neuritis; TM, transverse myelitis
